# Contribution of MylA in Conidiogenesis, Conidia Germination, and beta-Glucan Biosynthesis in *Aspergillus fumigatus*

**DOI:** 10.4014/jmb.2607.07015

**Published:** 2026-09-04

**Authors:** Ye-Eun Son, He-Jin Cho, Jae-Hyuk Yu, Hee-Soo Park

**Affiliations:** 1 School of Food Science and Biotechnology, Kyungpook National University, Daegu, 41566, Republic of Korea; 2 Department of Bacteriology, University of Wisconsin, Madison, WI 53706, USA; 3 Department of Integrative Biology, Kyungpook National University, Daegu, 41566, Republic of Korea; 4 Department of Advanced Bioconvergence, Kyungpook National University, Daegu, 41566, Republic of Korea

**Keywords:** β-glucan, Conidia, MYB-like protein, *Aspergillus fumigatus*, Germination

## Abstract

The conidia of *Aspergillus fumigatus* are the primary infectious propagules responsible for initiating invasive aspergillosis, and their development and physiology are closely regulated by numerous transcription factors. Our previous study demonstrated that the MYB-like transcription factor MylA plays a critical role in asexual sporulation and conidial viability in *Aspergillus nidulans*. However, the function of MylA in *A. fumigatus* has not yet been investigated. This study aimed to investigate the role of MylA, a member of the MYB-like transcription factor family, in conidiogenesis, conidial viability, cell wall integrity, and germination in *A. fumigatus*. Deletion of *mylA* resulted in reduced vegetative growth, abnormal conidiophore development, and a significant decrease in conidial production. Furthermore, Δ*mylA* conidia exhibited reduced trehalose accumulation, decreased viability, and increased sensitivity to heat and oxidative stress. Deletion of *mylA* also delayed conidial germination and impaired biofilm formation. Transcriptomic analysis of conidia identified approximately 2,200 differentially expressed genes, including those involved in β-glucan biosynthesis. Notably, deletion of *mylA* significantly upregulated the expression of *fks1* and *gel1*, resulting in increased β-glucan accumulation in the conidial cell wall. Collectively, these findings demonstrate that MylA is a key regulator of conidiogenesis, conidial viability, germination, and β-glucan biosynthesis in *A. fumigatus*.

## Introduction

*Aspergillus fumigatus* is a saprophytic fungus belonging to the phylum Ascomycota and is recognized as one of the most significant opportunistic human fungal pathogens [1]. *A. fumigatus* is commonly found in soil and decaying organic matter, where it produces airborne asexual spores (conidia) that are widely dispersed in both indoor and outdoor environments [2]. In healthy individuals, inhaled conidia are efficiently eliminated by the host immune system. However, in immunocompromised patients, the conidia can reach the lungs, where they germinate under favorable conditions and develop into invasive hyphae, ultimately causing invasive aspergillosis [3]. In addition to invasive aspergillosis, *A. fumigatus* is known to cause other pulmonary diseases, such as allergic bronchopulmonary aspergillosis (ABPA) and chronic pulmonary aspergillosis (CPA) [4, 5]. Along with *Candida albicans* and *Cryptococcus neoformans*, *A. fumigatus* is included in the World Health Organization (WHO) Fungal Priority Pathogens List (FPPL), highlighting its significance as a major human fungal pathogen [6]. Furthermore, treatment options for *A. fumigatus* infections remain limited, and the increasing prevalence of resistance to currently available antifungal agents has created an urgent need for the development of novel antifungal therapeutics [7].

The conidia of *A. fumigatus* possess a cell wall that is more resistant than that of vegetative hyphae, enabling them to survive in harsh natural environments as well as within the hostile conditions encountered in the human host [8, 9]. The fungal cell wall is primarily composed of chitin and β-glucan, whereas the outermost layer of conidia is enriched in chitin, β-glucan, melanin, and hydrophobins, which contribute to spore protection and immune evasion [9]. The composition of the cell wall and the acquisition of stress tolerance are closely regulated by multiple transcription factors [10]. Among these, the velvet family proteins VelB and VosA are well-characterized conidium-specific transcription factors that play key roles in regulating spore viability, maturation, and stress resistance across *Aspergillus* species [11]. Our recent study demonstrated that Myb-like protein A (MylA), a member of the MYB-like transcription factor family in *A. nidulans*, regulates conidial germination and stress tolerance, suggesting that MYB-like transcription factors contribute to the maintenance of conidial fitness [12].

The Myeloblastosis (MYB)-like proteins are characterized by the presence of a conserved MYB DNA-binding domain containing a helix-turn-helix (HTH) motif [13]. MYB-like proteins play various biological roles across eukaryotes [14]. In plants, they regulate morphogenesis, metabolism, and responses to various environmental stresses [15, 16]. In vertebrates, MYB proteins control the expression of cyclins and cyclin-dependent kinases (CDKs) involved in cell cycle progression and have been implicated in the development of numerous diseases, including cancer [17, 18]. In fungi, MYB-like proteins have been reported to regulate conidial germination in *Ashbya gossypii* [19] and *Fusarium graminearum* [20], as well as asexual and sexual development in *A. nidulans* [12]. In *Magnaporthe oryzae*, *Mo*Myb8 has been shown to contribute to stress tolerance [21]. Furthermore, in *Aspergillus sydowii*, the MYB transcription factors *As*Myb1 and *As*Myb3 regulate emodin biosynthesis, stress responses, and fungal development [22, 23]. Although several MYB-like proteins have been functionally characterized in these filamentous fungi, including *A. nidulans* and *A. sydowii* [22, 23], their biological roles in *A. fumigatus* remain largely unknown. To address this gap in the literature, this study investigated the function of MylA, a member of the MYB-like transcription factor family, in *A. fumigatus*. Through comprehensive phenotypic and transcriptomic analyses, this study demonstrates that MylA plays critical roles in conidiogenesis, conidial germination, and stress responses in this important opportunistic fungal pathogen.

## Materials and Methods

### Strains, Media and Culture Conditions

The *A. fumigatus* strains used in this study are listed in Table 1. The *A. fumigatus* AF293 strain was used as the wild-type strain, and the *A. fumigatus* AF293.1 strain was used as the parental strain for constructing deletion mutants. All *A. fumigatus* strains were cultured on liquid or solid minimal media with 1% glucose and 0.1 % yeast extract (MMY) [24]. For plasmid construction, *Escherichia coli* DH5α was cultured in Luria–Bertani medium supplemented with 100 μg/mL of ampicillin (Thermo Scientific, USA).

### Phylogenetic Analyses of MylA

To construct the phylogenetic tree, homologs and orthologs of MylA were identified using the Basic Local Alignment Search Tool (BLAST) developed by the National Center for Biotechnology Information (NCBI). These protein sequences were aligned using the CLUSTAL W algorithm in MEGA 12, and the phylogenetic tree was generated with CLC Sequence Viewer 8 using the neighbor-joining method based on the Jukes–Cantor model; bootstrap = 1,000 replicates) [25].

### Construction of Deletion And Complemented Strains

To generate the *mylA* deletion strain (Δ*mylA*), the double-joint PCR (DJ-PCR) method and PEG-mediated transformation were used [26, 27]. Oligonucleotides used in this study are listed in Table 2. To generate the deletion construct, the 5′ and 3′ regions of *mylA* were amplified using the primer sets OHS1906: OHS1907 and OHS1972: OHS1973, with *A. fumigatus* AF293 genomic DNA (gDNA) as the template. The *pyrG* marker was amplified using the primer set OHS2008: OHS2009, with *A. nidulans* FGSC4 gDNA as the template. The three PCR fragments were linked with OHS1909: OHS1910. The final construct was introduced into the *A. fumigatus* AF293.1, a *pyrG*^-^ strain. All transformants were confirmed by PCR and restriction enzyme mapping analyses.

To generate the *mylA* complemented strains, the promoter and open reading frame of the *mylA* gene were amplified using primers OHS1974: OHS1975, digested with PstI, and inserted into pYES1 [24]. The cloned plasmid pYE16.1 was introduced into the recipient Δ*mylA* strain TYE84.1 to yield TYE85.1. The *mylA* complemented strain was selected on MMY supplemented with pyrithiamine (1 μg/mL) and confirmed by PCR and quantitative reverse transcription PCR (qRT-PCR).

### Fungal Growth and Development

Each strain was point-inoculated onto solid MMY and incubated at 37°C for 5 days. Fungal colony growth was assessed by measuring colony diameter. Conidia were harvested using 0.02% Triton X-100 (Thermo Scientific), and conidia production was subsequently quantified using a hematocytometer under a Leica DM500 microscope (Leica Microsystems, Germany). All experiments were repeated at least three times.

### Conidial Trehalose Assay

The conidial trehalose content was determined as previously described [28]. Conidia from 2-day-old cultures grown on MMY solid media were collected using Miracloth. Conidia (2 × 10^8^) were resuspended in 200 μL of distilled water, incubated at 95°C for 20 min, and centrifuged at 13,000 rpm for 10 min. The supernatant (100 μL) was mixed with 100 μL of 0.2 M sodium citrate (pH 5.5) and incubated at 37°C for 8 h with or without trehalase (Sigma, USA). Trehalose content was quantified by measuring the amount of glucose released following trehalose hydrolysis by trehalase using a Glucose Assay Kit (Sigma). The measured glucose concentration was then converted to the corresponding trehalose content. These experiments were repeated three times.

### Conidial Viability Analysis

Conidia from cultures grown for 2, 7, 15, or 30 days were collected using distilled water. Approximately 100 conidia were spread onto solid MMY medium and incubated at 37°C for 2 days. Relative survival rates were calculated as the ratio of the number of viable colonies obtained from the 2-, 7-, 15-, or 30-day-old conidia to the number of viable colonies obtained from the 2-day control. The conidia viability assay was repeated three times.

### Conidial Stress Analysis

Conidia from 2-day-old cultures were collected and diluted to 10^3^ conidia/mL. For thermal stress, each sample was heated at 55°C for 30 or 60 min. For oxidative stress, each sample was incubated with 0.05 M H_2_O_2_ (Sigma) for 30 min. All samples were spread onto solid MMY medium and incubated at 37°C for 2 days. Relative survival rates were calculated as the ratio of the number of viable colonies in the treated sample to the number of viable colonies in the untreated control. The conidia stress response assay was repeated three times.

### Conidial Germination Analysis

The conidial germination assay was performed as described in a previous study [29]. Approximately 1 × 10^7^ conidia collected from 2-day-old cultures were spread onto solid MMY medium and incubated at 37°C. Germination rates were measured every hour and calculated as the ratio of the number of germinated conidia to the total number of conidia in each sample. The assay was repeated three times.

### Fungal Biofilm Assay

Fungal biofilm formation was assessed as previously described [30]. Conidia harvested from 2-day-old cultures were suspended in distilled water, and 2 × 10^5^ conidia/mL were inoculated into 150 μL of liquid minimal medium in each well of a 96-well plate. The plate was incubated at 37°C for 24 h. The medium was then removed, and the wells were washed three times with 200 μL of phosphate-buffered saline (PBS). Biofilms were stained with 100 μL of 0.1% (w/v) crystal violet for 10 min at room temperature, washed three times with PBS, and destained with 125 μL of ethanol. The destaining solution was transferred to a new 96-well plate, and biofilm biomass was quantified by measuring the absorbance at 570 nm. At least three technical replicates were included for each strain.

### Transcriptome Analysis

For transcriptomic analysis, conidia of *A. fumigatus* grown on MMY solid medium for 2 days were isolated, suspended, and filtered through Miracloth. RNA extraction was performed as previously described [29]. Briefly, total RNA was isolated using RiboEx^TM^ reagent (GeneAll Biotechnology, Republic of Korea) and purified using the RNeasy Mini Kit (Qiagen, Germany). The RNA libraries were prepared by Theragen Bio (Theragen Bio, Republic of Korea) and sequenced on the Illumina NovaSeq 6000 platform. The resulting FASTQ files were trimmed with Trimmomatic, and the filtered reads were annotaed with the *A. fumigatus* AF293 transcriptome using STAR v.2.3.0e software. Differential expressed genes (DEG) were identified and annotated using the DESeq2 method [31]. DEGs were screened with a fold change ≥ 2 and a *p*-value < 0.05, and Gene Ontology (GO) analysis was performed using R packages and FungiDB. Statistical significance was assessed using Fisher’s exact test, and the results were visualized using ggplot in R studio (version 4.2.2; https://www.R-project.org/). All RNA-seq experiments were performed using at least three biological replicates.

### qRT-PCR Analysis

qRT-PCR was performed as previously described [29]. Complementary DNA (cDNA) was synthesized using GoScript Reverse Transcriptase (Promega, USA), and the resulting cDNA was used as a template for qPCR. qPCR was performed using iTaq Universal SYBR Green Supermix (BioRad, USA) on a CFX96 Touch Real-Time PCR system (BioRad). The target-specific primer sets used in this study are listed in Table 2. The expression levels were calculated using the 2^−ΔΔCt^ method and the expression was normalized to that of β-actin, which was used as a control. All qPCR experiments were performed in triplicate.

### Conidial β-Glucan Analysis

Conidial β-glucan analysis was performed as previously described with modifications [12]. Conidia obtained from 2-day-old cultures were collected using Miracloth. Each sample (10^3^~10^4^ conidia) was resuspended in 25 μL and mixed with 25 μL of Glucatell^®^ reagent (Associates of Cape Cod, MA, USA) and incubated at 37°C for 30 min. To terminate the reaction, diazo reagent were added to all samples and the absorbance at 540 nm was measured. The assay was repeated three times.

### Statistical Analysis

All data are presented as mean ± standard deviation (SD), and one-way ANOVA was used to analyze statistical differences among WT, deletion mutants, and complemented strain. A *p*-value < 0.05 was considered statistically significant. All statistical analyses were performed using GraphPad Prism software (version 11; https://www.graphpad.com/).

## Results

### MylA is Required for Proper Asexual Development in *A. fumigatus*

In our previous study, we characterized MylA, a member of the MYB-like transcription factor family, in *A. nidulans*. The *mylA* gene is a conidia-specific gene and encodes a MYB-like transcription factor containing two imperfect MYB repeats, which plays essential roles in vegetative growth and asexual development [12].

To identify the *A. fumigatus* ortholog of *A. nidulans* MylA, we performed a homology search and identified the protein encoded by the Afu2g02350 gene. Further sequence analyses revealed that MylA is highly conserved among *Aspergillus* species (Fig. 1A). Domain analysis showed that the protein contains a conserved SANT/MYB DNA-binding domain, and structural analysis predicted the characteristic α-helical architecture of the MYB domain (Fig. 1B).

To investigate the biological function of MylA in *A. fumigatus*, we generated a *mylA* deletion mutant (Δ*mylA*) strain and a complemented strain (C′ *mylA*). Colony morphology and conidiation were examined by inoculating the strains onto MMY medium (Fig. 1C). Compared with the wild-type and complemented strains, the Δ*mylA* strain exhibited considerably reduced colony growth. Microscopic observation further revealed that the conidiophore heads of the Δ*mylA* strain were substantially smaller than those of the wild-type strains (Fig. 1C). Quantitative analysis confirmed that deletion of *mylA* significantly reduced both colony diameter and conidial production (Fig. 1D and 1E). These results demonstrate that MylA is required for normal vegetative growth and asexual development in *A. fumigatus*.

### MylA is Involved In Trehalose Biosynthesis, Conidial Viability, and Stress Tolerance

Based on our previous finding that MylA regulates conidial development and dormancy in *A. nidulans* [12], we examined the role of MylA in conidial physiology in *A. fumigatus*. We first measured trehalose content, which is closely associated with conidial stress tolerance. The Δ*mylA* mutant contained only approximately 40% of the trehalose level detected in conidia of the wild-type strain (Fig. 2A). We then assessed conidial viability. Conidia harvested from cultures grown for 2 or 7 days exhibited no significant difference in viability between the wild-type and Δ*mylA* strains. However, conidia collected from 15- or 30-day-old cultures showed a significant reduction in viability in the Δ*mylA* mutant compared with the wild-type strain (Fig. 2B), indicating impaired long-term conidial survival. To further evaluate the role of MylA in stress tolerance, we examined conidial sensitivity to thermal and oxidative stresses (Fig, 2C, 2D). The Δ*mylA* mutant conidia showed significantly greater sensitivity to both heat and oxidative stress than wild-type conidia. Collectively, these results indicate that MylA is required for trehalose accumulation during conidial maturation and plays critical roles in maintaining conidial dormancy and stress tolerance in *A. fumigatus*.

### MylA is Essential For Normal Conidial Germination and Biofilm Formation

To investigate the role of MylA in conidial germination, conidia from the wild-type and Δ*mylA* strains were inoculated into MMY medium, and the conidial germination rate was monitored. Germination was assessed from 3 to 10 h post-inoculation. At 7 h, the Δ*mylA* mutant exhibited a significantly lower germination rate than the wild-type strain (Fig. 3A, 3B). Notably, by 9 h post-inoculation, the germination rate of the Δ*mylA* mutant increased to a level comparable to that of the wild-type strain (Fig. 3B). However, the germ tubes of the Δ*mylA* mutant remained significantly shorter than those of the wild-type strain (Fig. 3A). These findings indicate that deletion of *mylA* delays conidial germination and impairs subsequent germ tube elongation in *A. fumigatus*.

Because the Δ*mylA* strain exhibited delayed conidial germination and impaired germ tube elongation, we examined whether these defects affected biofilm formation. Crystal violet staining demonstrated that biofilm biomass was significantly reduced in the Δ*mylA* mutant compared with the wild-type and complemented strains (Fig. 3C). These results suggest that MylA contributes to biofilm development, at least in part, by facilitating conidial germination and hyphal growth.

### MylA Contributes to β-Glucan Biosynthesis in *A. fumigatus* Conidia

As mentioned above, MylA plays an important role in conidial development and physiology. To investigate the molecular basis of these functions, we performed transcriptomic analysis. Total RNA was isolated from wild-type and Δ*mylA* conidia, and RNA-seq analysis was performed. Transcriptomic analysis identified a total of 2,262 DEGs between the two strains (Fig. 4A). GO enrichment analysis revealed that the upregulated genes were primarily associated with the ncRNA processing, organic substance metabolic process, and primary metabolic process, whereas the downregulated genes were significantly enriched in protein N-linked glycosylation, alkaloid biosynthetic process, and alkaloid metabolic process (Fig. 4B).

Among the DEGs, we observed notable changes in the expression of genes involved in β-glucan biosynthesis. Specifically, the expression levels of *fks1* (1,3-beta-glucan synthase), *gel1* (1,3-beta-glucanosyltransferase 1), *gel4* (1,3-beta-glucanosyltransferase 4), and *bgt1* (beta-1,3-glucanosyltransferase) were significantly increased in the Δ*mylA* mutant compared with the wild-type strain (Fig. 5A). To validate the RNA-seq data, the mRNA levels of *fks1* and *gel1* were further examined by qRT-PCR, which yielded expression patterns consistent with the transcriptomic data (Fig. 5B). To determine whether these transcriptional changes affected β-glucan accumulation, we quantified the β-glucan content in conidia. As shown in Fig. 5C, Δ*mylA* conidia contained approximately a twofold higher level of β-glucan than the conidia of the wild-type and complemented strains. Collectively, these findings demonstrate that MylA plays an important role in regulating β-glucan biosynthesis in *A. fumigatus* conidia.

## Discussion

MYB-like proteins are key regulatory molecules involved in fungal growth, development, secondary metabolism, and stress responses across a wide range of fungi [21, 32]. In *A. nidulans*, four MYB-like proteins have been characterized: AdaB [33], which is involved in chromatin remodeling; FlbD [34], an upstream developmental activator; CicD [35], a transcriptional activator of cichorine biosynthesis; and MylA [12]. These proteins primarily regulate fungal development, and some also contribute to secondary metabolism. In *A. fumigatus*, the function of the Myb transcription factor MybA has been characterized [36, 37]; however, the functions of MYB-like proteins have not been investigated. Therefore, this study provides the first functional characterization of a MYB-like protein in *A. fumigatus*.

Comparative analysis of MylA function in *A. fumigatus* and *A. nidulans* provides valuable insights into the conserved biological roles of this MYB-like protein. In both species, deletion of *mylA* resulted in reduced colony diameter and significantly decreased conidial production (Fig. 1). Notably, the size of the conidial head was also considerably reduced in the *mylA* deletion mutants of both fungi (Fig. 1C). These conserved phenotypic defects suggest that MylA plays a fundamental role in regulating asexual development across *Aspergillus* species. Therefore, MylA is likely to be an evolutionarily conserved regulator required for proper conidiophore development and efficient conidiation in the genus *Aspergillus*.

An important finding from both the previous study in *A. nidulans* [12] and the present study in *A. fumigatus* is that MylA plays a critical role in conidiogenesis. After conidia are formed, they undergo maturation, enter dormancy, and subsequently germinate under appropriate environmental conditions. Our results demonstrate that deletion of *mylA* affects multiple stages of the conidial life cycle. During conidial maturation, trehalose biosynthesis is essential for spore longevity and stress tolerance [38]. In the *mylA* deletion mutant, the expression of trehalose biosynthetic genes was significantly reduced, accompanied by a notable decrease in trehalose accumulation in conidia in both species (Fig. 2). Additionally, *mylA* mutant conidia in both species exhibited increased sensitivity to multiple environmental stresses and reduced spore viability, indicating impaired conidial maturation and maintenance. Conidial germination was also dysregulated in the absence of *mylA*, suggesting that MylA is required not only for establishing dormancy but also for the proper initiation of germination. Collectively, these findings indicate that MylA is a key regulator of the entire conidial life cycle, coordinating conidial maturation, dormancy, viability, stress resistance, and germination.

Our transcriptomic analysis revealed altered expression of several central developmental regulators of asexual development in the Δ*mylA* conidia. Specifically, *brlA* (log2FC= -1.12) and *abaA* (log2FC= -1.49) were significantly downregulated, whereas *wetA* showed only a marginal decrease (log2FC = -0.33). These transcriptional changes may contribute to the defects in conidiogenesis. In contrast, *vosA* and *velB*, which encode well-established master regulators of conidial maturation and dormancy [39-41], showed increased expression, with *vosA* being significantly upregulated (log2FC= 1.05) and *velB* exhibiting an increasing trend (log2FC= 0.99), although the latter was not statistically significant. Because VosA negatively regulates *brlA* expression, increased *vosA* expression may contribute to the reduced expression of *brlA* and the consequent defects in conidiogenesis. Furthermore, deletion of *vosA* has been reported to accelerate conidial germination, suggesting that elevated *vosA* expression may also contribute to the altered germination phenotype observed in the Δ*mylA* strain. Given these broad regulatory functions, further studies are needed to determine how MylA interacts with key developmental regulators, including BrlA, AbaA, and other spore-specific regulators,. Elucidating the genetic and molecular relationships between MylA and these regulators will provide a comprehensive understanding of the mechanisms governing conidiogenesis in *Aspergillus* species.

Another finding in the *mylA* deletion mutant was the increased accumulation of β-glucan in conidia (Fig. 5). This phenotype was consistent with the transcriptomic results, which revealed the upregulation of several genes involved in β-glucan biosynthesis in the *mylA* mutants of *A. nidulans* and *A. fumigatus*. These findings indicate that MylA influences β-glucan biosynthesis. However, these data cannot support that MylA directly regulates the transcription of β-glucan biosynthetic genes. Therefore, future studies, such as chromatin immunoprecipitation followed by sequencing, will be required to determine whether MylA directly binds to and regulates genes involved in β-glucan biosynthesis. In *A. nidulans*, the absence of *mylA* also resulted in increased expression of genes involved in the biosynthesis of hydrophobins, DHN-melanin, and galactomannan, all of which are major components of the fungal cell wall [12]. Similarly, RNA-seq analysis of *A. fumigatus* conidia showed increased expression of the hydrophobin genes *rodA* and *rodE* (Fig. 4), as well as the melanin biosynthetic genes *arp2* and *pksP*, in the *A. fumigatus* *mylA* deletion mutant. Collectively, these findings suggest that MylA acts as a regulator of cell wall integrity and plays an important role in maintaining cell wall homeostasis during conidial development. Future studies should determine whether MylA directly regulates the mRNA expression of genes involved in spore wall integrity or modulates their expression indirectly through upstream developmental regulatory networks.

Our transcriptome analysis revealed significant enrichment of genes involved in ncRNA processing and ribosome biogenesis in the Δ*mylA* mutant (Fig. 4). Specifically, many of the upregulated genes encode proteins involved in distinct stages of ribosome biogenesis, including early pre-rRNA processing (MRD1, NOP9, RRP36, UTP10, and NOC4), RNA remodeling by DEAD-box RNA helicases (HAS1, DBP8, and DBP9), snoRNP-mediated rRNA modification (CBF5 and NOP58), and large ribosomal subunit assembly (ERB1 and SPB1). These changes indicate a coordinated transcriptional upregulation of genes involved in ribosome biogenesis. Although proper ribosome biogenesis is essential for fungal growth and morphogenesis [42], the Δ*mylA* mutant exhibited delayed conidial germination, impaired germ tube elongation, reduced colony growth, and defective biofilm formation. Thus, the coordinated upregulation of ribosome biogenesis-related genes was unexpected. Although the biological significance of these transcriptional changes remains unclear, they may reflect alterations in cellular physiology associated with the impaired growth of the Δ*mylA* mutant. One possible explanation is that the increased expression of these genes represents a compensatory response to impaired growth; however, this response appears insufficient to restore normal growth and development. Further studies are required to determine whether these transcriptional changes contribute directly to the observed phenotypes or represent secondary consequences of the growth defects.

*A. fumigatus* conidia rely on a robust cell wall structure and biofilm formation to survive under diverse environmental conditions and to establish infection within the host [43]. In the present study, the Δ*mylA* mutant exhibited increased sensitivity to thermal and oxidative stresses, reduced conidial viability, and impaired biofilm formation, indicating that MylA plays an important role in maintaining conidial fitness (Figs. 2 and 3). These phenotypic defects suggest that MylA may also contribute to the pathogenicity of *A. fumigatus*, as stress tolerance, conidial viability, and biofilm formation are key factors associated with fungal survival and virulence. Although the present study did not directly investigate the role of MylA in virulence, future studies using immunocompromised mouse models will be necessary to determine the contribution of MylA to the pathogenesis of *A. fumigatus*.

In conclusion, through our previous study in *A. nidulans* [12] and the present study in *A. fumigatus*, we characterized the biological functions of MylA, a member of the MYB-like protein family, and demonstrated that it plays critical roles in fungal growth and asexual development. Notably, our findings reveal that MylA functions as a key regulator throughout the conidial life cycle, influencing conidial maturation, dormancy, germination, stress tolerance, viability, and cell wall homeostasis. Consistent with these phenotypic changes, deletion of *mylA* resulted in significant alterations in the expression of genes associated with trehalose metabolism, cell wall biosynthesis, and conidial development, suggesting that MylA coordinates multiple regulatory pathways required for optimal conidial physiology. Future studies should focus on elucidating the molecular mechanisms underlying MylA function, including the identification of its direct downstream targets and regulatory partners. Investigating the functional relationship between MylA and other spore-specific regulators, such as VosA, VelB, and WetA, will provide important insights into the genetic network governing conidial development in *Aspergillus*. These studies will advance our understanding of the comprehensive regulatory framework regulating conidiation in *Aspergillus* species.

## Figures and Tables

**Fig. 1:**
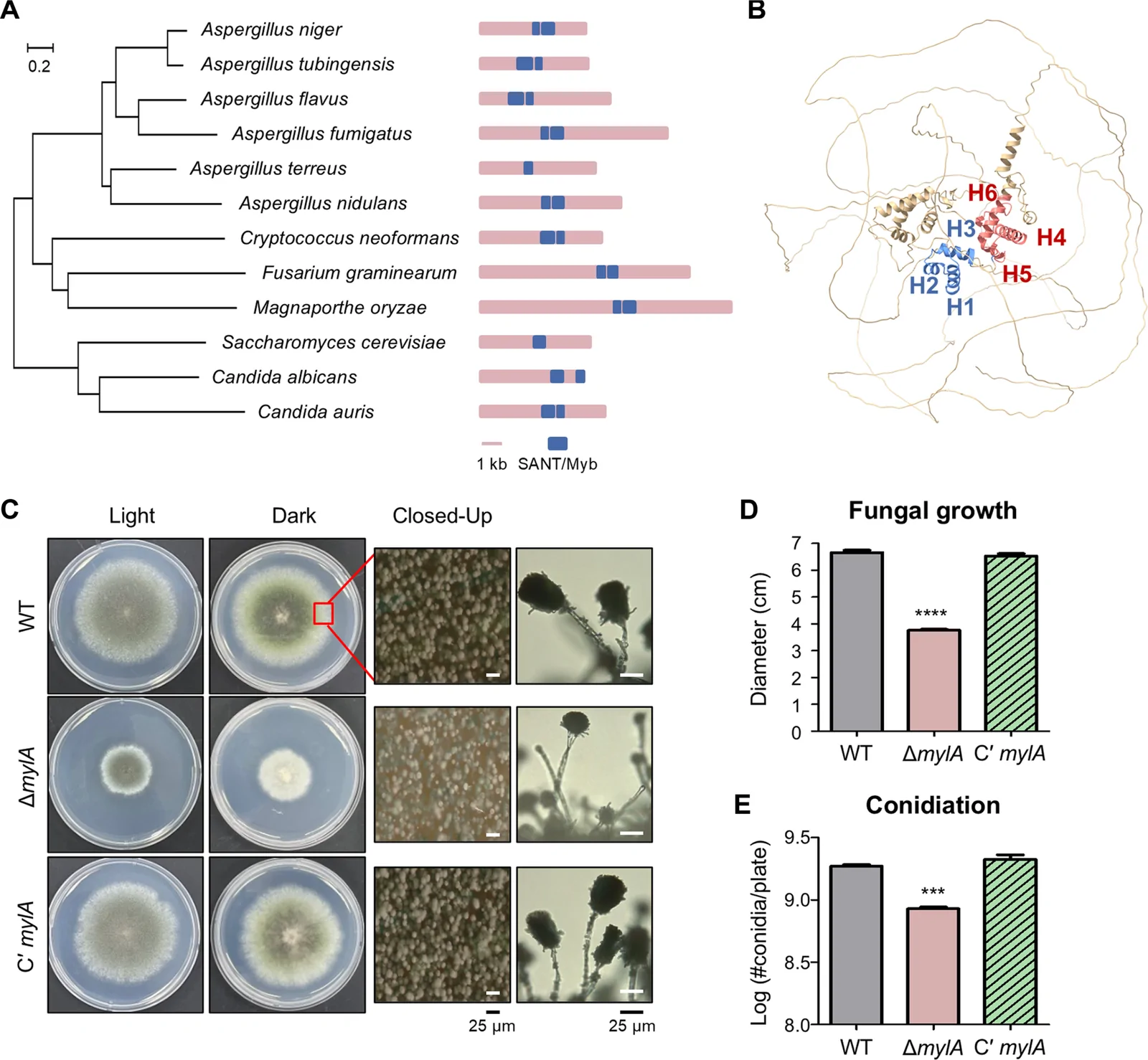
Functions of MylA in fungal growth and conidiation. (**A**) Phylogenetic tree and domain analysis of MylA homologous or orthologues in fungi. (**B**) Secondary structure of MylA in *A. fumigatus*, predicted using AlphaFold3. (**C**) Phenotypes of wild-type (WT), Δ*mylA*, and complemented (C′ *mylA*) strains grown on solid MMY medium at 37°C for 5 days under dark or light conditions. The right panels show magnified images of the center of the colonies and the conidiophore morphology of each strain (Bar = 25 μm). (**D**) Fungal colony diameters of WT, Δ*mylA*, and C′ *mylA* strains shown in panel C (****p < 0.0001). (**E**) Number of conidia of WT, Δ*mylA*, and C′ *mylA* strains shown in panel C (***p < 0.001)

**Fig. 2:**
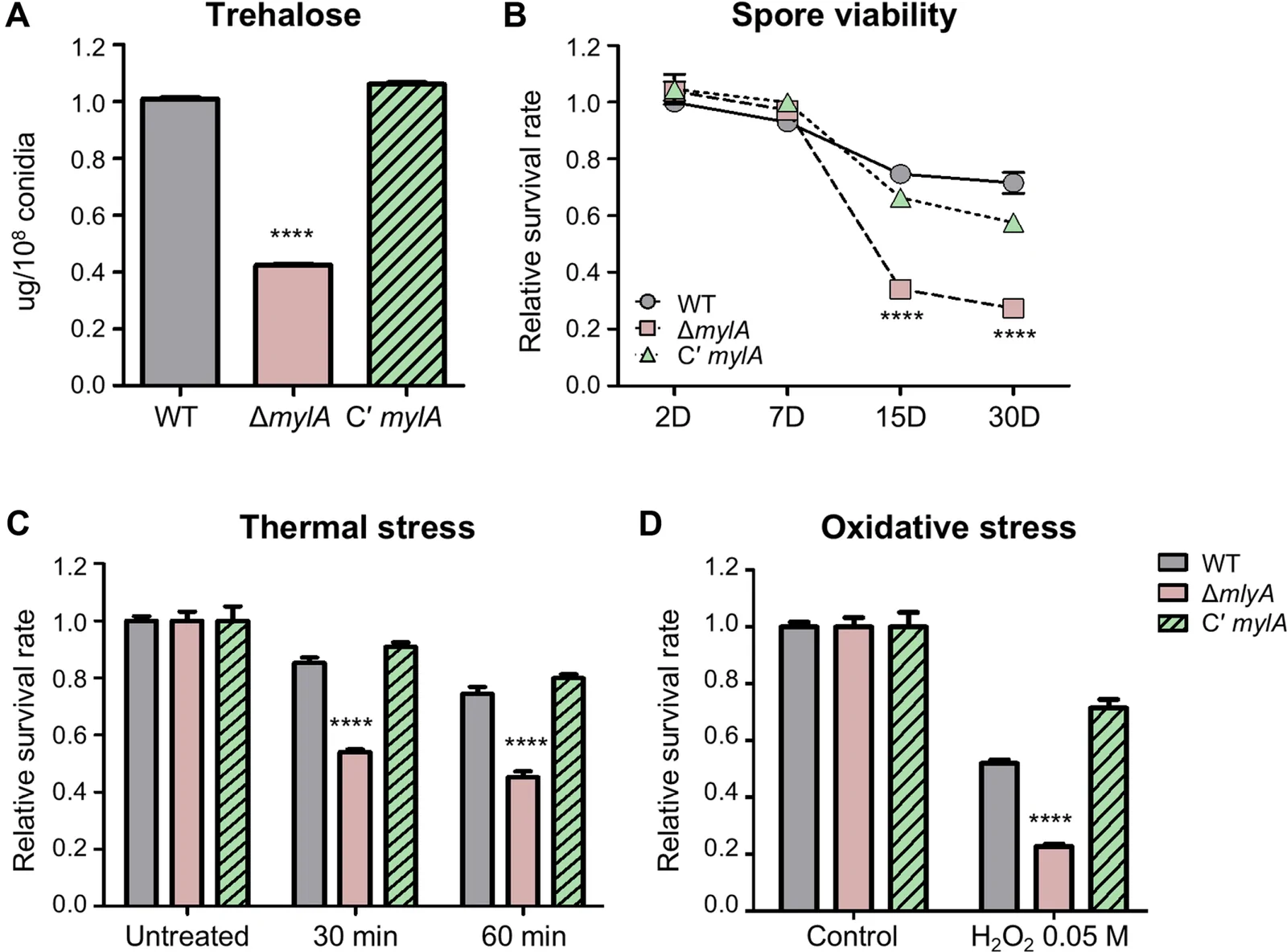
Roles of MylA in conidia. (**A**) Trehalose content in WT, Δ*mylA*, and C′ *mylA* conidia (****p < 0.0001). (**B**) Conidial viability of WT, Δ*mylA*, and C′ *mylA* strains (****p < 0.0001). (**C**) Relative spore survival rates of WT, Δ*mylA*, and C′ *mylA* strains against thermal stress (55°C for 30 min, ****p < 0.0001). (**D**) Relative spore survival rates of WT, Δ*mylA*, and C′ *mylA* strains against oxidative stress (0.05 M H_2_O_2_ for 30 min, ****p < 0.0001).

**Fig. 3:**
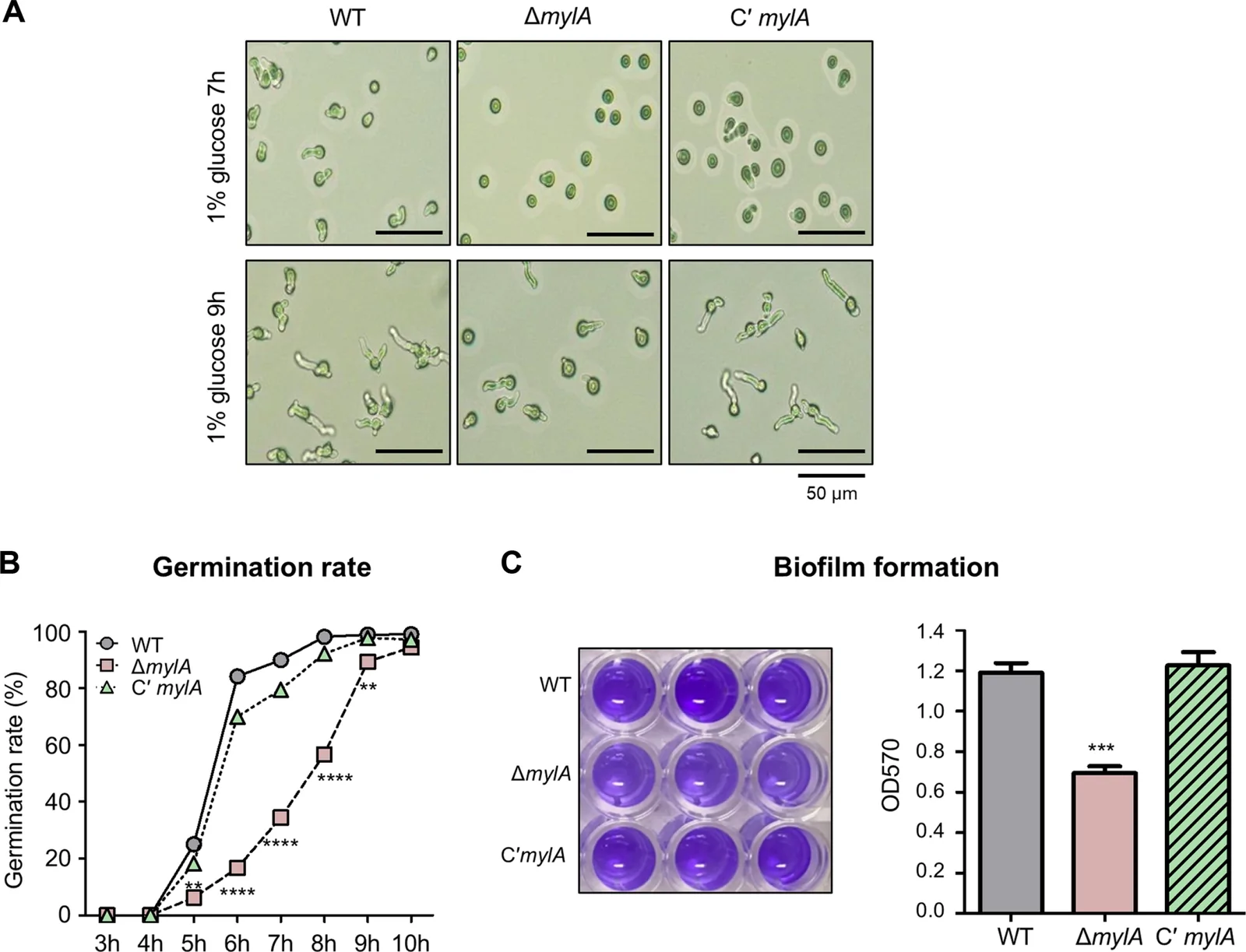
Effect of MylA on conidial germination. (**A**) Images of conidial gemination of WT, Δ*mylA*, and C′ *mylA* after 7 or 9 h of incubation (scale bar = 50 μm). (**B**) Germination rates of WT, Δ*mylA*, and C′ *mylA* conidia (**p < 0.01, ***p < 0.001, ****p < 0.0001). (**C**) Biofilm formation by WT, Δ*mylA*, and C′ *mylA* (***p < 0.001)

**Fig. 4:**
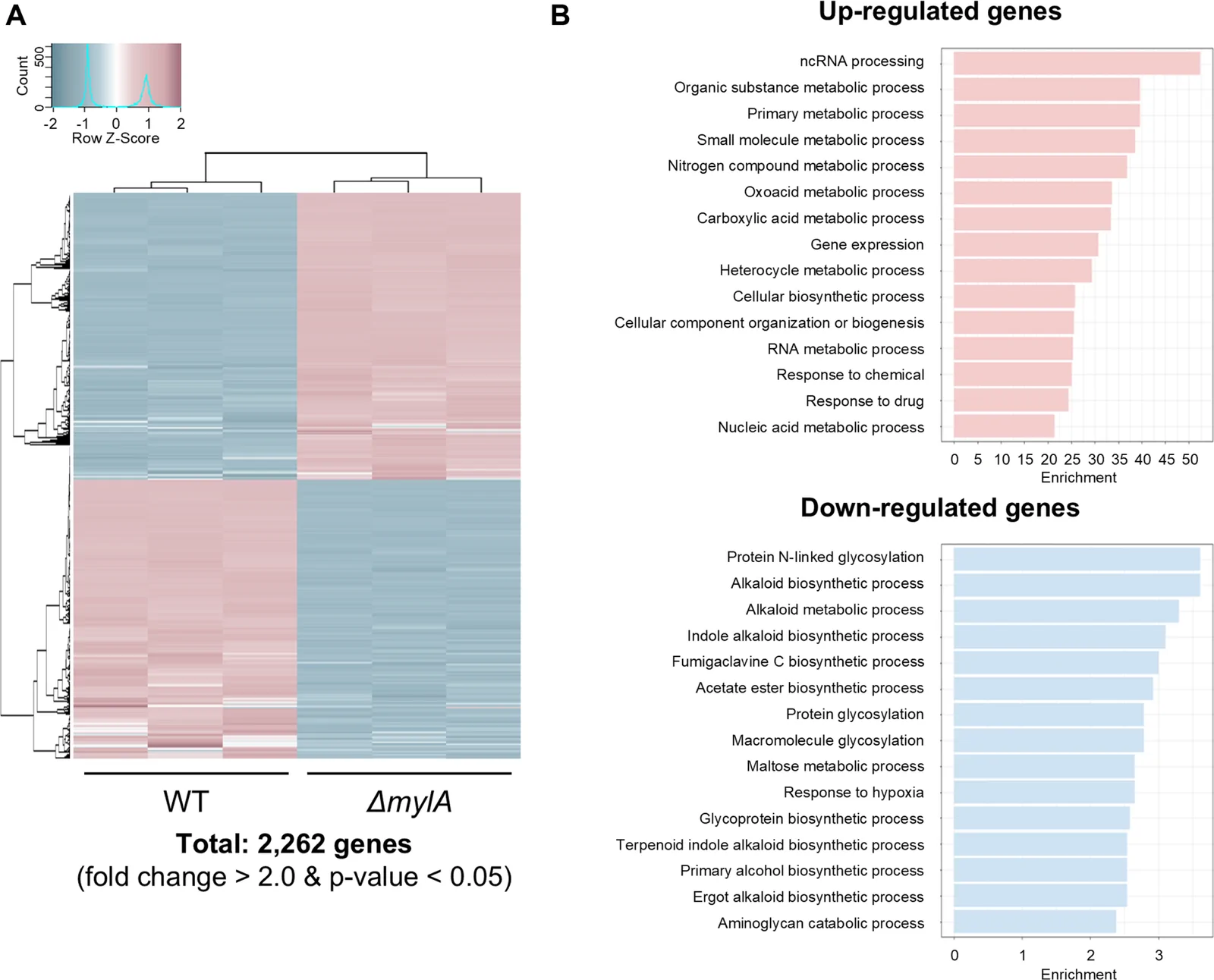
Transcriptomic analysis of Δ*mylA* C′*mylA*. (**A**) Heat map showing differentially expressed genes between WT and Δ*mylA* conidia (fold change > 2.0; adjusted *p*-value < 0.05). (**B**) GO analysis of upregulated and downregulated genes in Δ*mylA* conidia

**Fig. 5:**
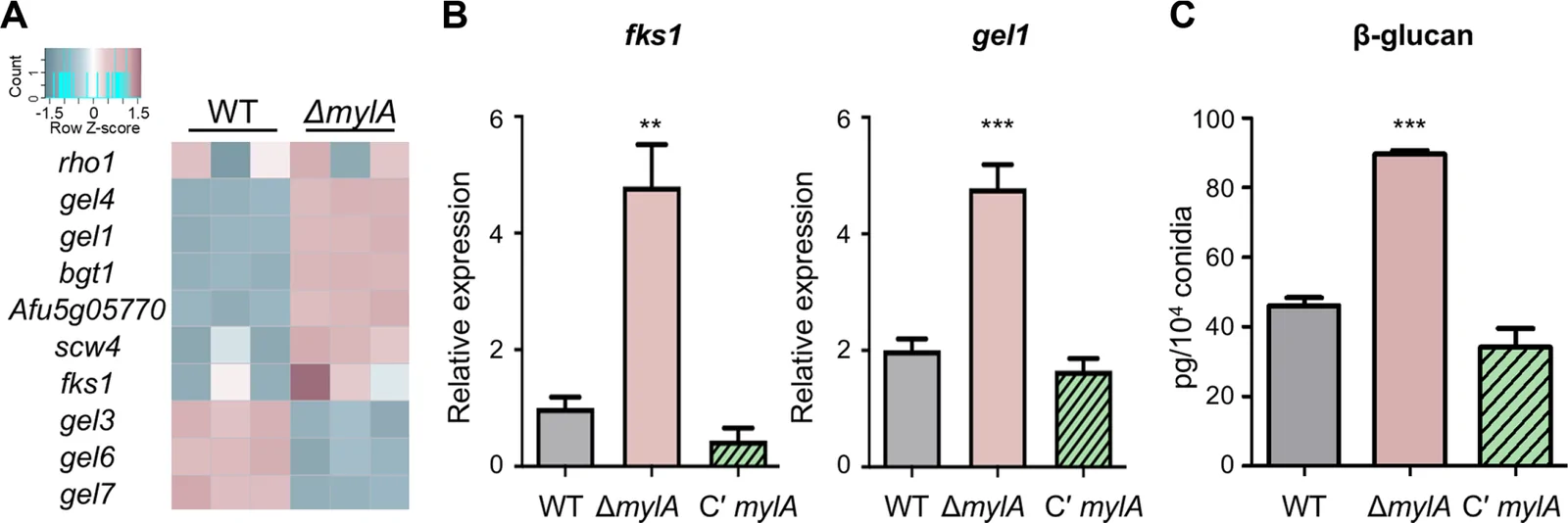
Role of MylA in *A. fumigatus* β-glucan biosynthesis. (**A**) Heat map showing differentially C'*mylA* genes associated with β-glucan biosynthesis between WT and Δ*mylA* conidia. (**B**) mRNA levels of *fks1* and *gel1* in WT, Δ*mylA*, and C′*mylA* conidia (**p < 0.01, ***p < 0.001). (**C**) β-glucan content in WT, Δ*mylA*, and C′ *mylA* conidia (***p < 0.001).

**Table 1:** *Aspergillus* strains used in this study.

Strain	Relevant genotype	References
AF293	*A. fumigatus* wild type	[44]
AF293.1	pyrG1	[45]
TYE84.1–2	*pyrG1*; *ΔmylA*::*AnipyrG*^+^	This study
TYE85.1	*pyrG1*; *ΔmylA*::*AnipyrG*^+^; *mylA*(*p*)::*mylA*::FLAG_4x_::*ptrA*^R^	This study

^a^Fungal Genetic Stock Center

**Table 2:** Oligonucleotides used in this study.

Name	Sequence (5′ → 3′)^a^	Purpose
OHS2008	TCATCCATGGTGTCCTCGTC	*AnipyrG*_Marker_F
OHS2009	CTGGAATCAGTGGAGCGAAC	*AnipyrG*_Marker_R
OHS1906	GCCAGTCAAGCCATTGATCC	5′ *mylA* DF
OHS1907	*TTTGTAGGCTTTGGGCTGTTCACAA* TGCGAGTCAGTAGCTCTCG	3′ *mylA* with *AnipyrG* tail
OHS1972	*CTGATCTACCCCTTGGAACGCAGCA* AGATCTCGGCTCGACACG	5′ *mylA* with *AnipyrG* tail
OHS1973	CACCTGTACGAGCGAATGCT	3′ *mylA* DR
OHS1910	GATTACGCCGCAATCGCC	5′ *mylA* NF
OHS1909	CCGTGATGCGAAACACCA	3′ *mylA* NR
OHS1912	AACGTAAAGCTCAGCAACCC	5′ *mylA* RT_F
OHS1913	GGTATCGGCTTCTGAATCGC	3′ *mylA* RT_R
OHS1974	AATT **CTGCAG** CAATCGCCTCATGTGCTGCT	5′ *mylA* with promoter and *Pst*I
OHS1975	AATT **CTGCAG** GGAATTTTTCCCCTTTGACACGGG	3′ *mylA* with *Pst*I
OHS2512	TCTGGGTGCTTTCTGGTTCT	5′ *fks1* RT_F
OHS2513	GCGGACCAAATCCTGTAACC	3′ *fks1* RT_R
OHS2514	CTGACTGTCCCTAGCCTGAC	5′ *gel1* RT_F
OHS2515	GACGGTGGACAGGAGTGTAA	3′ *gel1* RT_R

^a^Tail sequences are shown in italics
